# Constructing Japanese MeSH term dictionaries related to the COVID-19 literature

**DOI:** 10.5808/gi.21012

**Published:** 2021-09-30

**Authors:** Atsuko Yamaguchi, Terue Takatsuki, Yuka Tateisi, Felipe Soares

**Affiliations:** 1 Graduate School of Integrative Science and Engineering, Tokyo City University, Tokyo 158-8557, Japan; 2 Database Center for Life Science, Research Organization of Information and Systems, Kashiwa 277-0871, Japan; 3 National Bioscience Database Center, Japan Science and Technology Agency, Chiyoda, Tokyo 102-8666, Japan; 4 Computer Science Department, The University of Sheffield, Western Bank, Sheffield S10 2TN, UK

**Keywords:** COVID-19, Japanese, medical vocabulary, MeSH

## Abstract

The coronavirus disease 2019 (COVID-19) pandemic has led to a flood of research papers and the information has been updated with considerable frequency. For society to derive benefits from this research, it is necessary to promote sharing up-to-date knowledge from these papers. However, because most research papers are written in English, it is difficult for people who are not familiar with English medical terms to obtain knowledge from them. To facilitate sharing knowledge from COVID-19 papers written in English for Japanese speakers, we tried to construct a dictionary with an open license by assigning Japanese terms to MeSH unique identifiers (UIDs) annotated to words in the texts of COVID-19 papers. Using this dictionary, 98.99% of all occurrences of MeSH terms in COVID-19 papers were covered. We also created a curated version of the dictionary and uploaded it to PubDictionary for wider use in the PubAnnotation system.

## Introduction

An enormous number of research papers about the coronavirus disease 2019 (COVID-19) pandemic has recently been published, and knowledge about COVID-19 is very frequently updated through research articles. As a result of these rapid updates, people's understanding easily becomes outdated. It may be especially difficult for people who are not English speakers to understand research papers about COVID-19 because they include many English medical terms. To share up-to-date knowledge among Japanese speakers from COVID-19 research papers written in English, a Japanese dictionary of medical terms related to COVID-19 is required.

Due to the time constraints of the 7th Biomedical Linked Annotation Hackathon (BLAH7) hackathon, we focused on the Medical Subject Headings (MeSH) unique identifiers (UIDs) annotated in LitCovid [1], which is a curated literature hub providing centralized access to relevant articles in PubMed. Our goal of the hackathon was to construct a dictionary with an open license that has maps of MeSH UIDs in LitCovid and Japanese terms with as many curated terms as possible.

Existing Japanese translations of MeSH terms were not suitable for our purpose. There is a translation of MeSH into Japanese (MeSHJPN) included in the Unified Medical Language Systems (UMLS), provided by the Japan Medical Abstracts Society (JAMAS). The translation is based on the JAMAS Japanese Medical Thesaurus (JJMT). The latest version of JJMT was released in 2019, and corresponds to MeSH 2018, but MeSHJPN is based on the previous version (2014). This means that both dictionaries are older than the onset of the COVID-19 pandemic. In addition, JJMT is not downloadable, and MeSHJPN has a Category 3 License Restriction (https://uts.nlm.nih.gov/uts/license/license-category-help.html#category3) that prohibits the incorporation of the dictionary into any publicly accessible computer-based information systems. Thus, those resources are not suitable for sharing knowledge in an open manner. There are other translations of MeSH into Japanese but, as reported in 5th Biomedical Linked Annotation Hackathon (BLAH5) [2], they have similar problems.

Therefore, we tried to construct a dictionary with an open license by using datasets that are freely available. By using six datasets with open licenses and adding some Japanese terms manually, we constructed a dictionary with a map from MeSH UIDs to Japanese terms.

## Methods

As described above, we focused on the MeSH UIDs annotated in LitCovid. We first extracted the MeSH UIDs from LitCovid and sorted them according to the number of occurrences. Next, we obtained pairs of MeSH UIDs and the corresponding Japanese terms using the following six datasets that are freely available.

### Wikidata

Wikidata (https://wikidata.org/) is a free and open knowledge base that includes MeSH UIDs and their multilingual labels. Wikidata provides a SPARQL endpoint with a graphical user interface (https://query.wikidata.org/). Using the SPARQL endpoint, we obtained pairs of MeSH UIDs and Japanese labels.

### Japan Science and Technology Agency thesaurus headwords from the MeCab user dictionary for science technology terms

The National BioScience Database Center provides dictionaries for the Japanese morphological analyzer MeCab, based on life science-related vocabulary from the Japan Science and Technology Agency (JST) thesaurus, 2015 edition. Although it is not based on the latest version of the JST thesaurus, which is for profit, the user dictionaries based on the 2015 edition are available through a Creative Commons Attribution-Share Alike 4.0 International (CC-BY-SA 4.0). One of the dictionaries consists of terms that can be assigned MeSH UIDs via the Interlinking Ontology of Biological Concepts [3] (https://bioportal.bioontology.org/ontologies/IOBC).

The dictionary can be downloaded from Life Science Database Archive (https://dbarchive.biosciencedbc.jp/en/mecab/data-2.html). We used the correspondences of Japanese terms and MeSH UIDs from the dictionary.

### Human Phenotype Ontology

The Human Phenotype Ontology (HPO) [4] is a standardized vocabulary of phenotypic abnormalities encountered in human disease. HPO terms have links to MeSH UIDs, and Japanese labels for HPO terms are freely available at https://github.com/ogishima/HPO-japanese. Therefore, we were able to obtain Japanese terms for the MeSH UIDs included in the HPO.

### Kyoto Encyclopedia of Genes and Genomes (KEGG) disease and KEGG drug

The KEGG is a collection of databases dealing with genomes, biological pathways, diseases, drugs, and chemical substances [5]. Although the KEGG database is not public, a subset of the databases about medical science, including KEGG Disease and KEGG Drug, is freely available from https://dbarchive.biosciencedbc.jp/en/kegg-medicus/download.html with a CC-BY-SA 4.0 license. These include both English and Japanese labels for the entries. For each MeSH term, if the dictionary included exactly the same English word as a label for an entry, we obtained the Japanese label for the same entry for the MeSH UID.

### 6th Biomedical Linked Annotation Hackathon (BLAH6) dictionary

Yamada and Tateisi [6] created a dictionary of MeSH UIDs and Japanese terms at the BLAH6 hackathon by using the open-japanese-mesh script (https://github.com/roy29fuku/open-japanese-mesh) from two glossaries: MeSpEn by Barcelona Supercomputing Center (https://temu.bsc.es/mespen/) and MEDUTX (an English-Japanese dictionary by Kitasato University available from Asia-Pacific Association for Machine Translation, https://aamt.info/wp-content/uploads/2019/06/medutx1.05.zip) as reported previously [6]. We obtained the Japanese terms for MeSH IDs from the dictionary.

We obtained pairs of MeSH UIDs and Japanese terms independently for each dataset. Next, we divided the MeSH UIDs into two groups according to whether they had at least 50 occurrences or fewer than 50 occurrences. For the MeSH UIDs in the first group, if two or more different Japanese terms are assigned by the dictionaries, we selected one Japanese term manually. If no Japanese term was assigned to a MeSH UID in the first group, three native Japanese speakers who have been researchers in the life-sciences domain for more than 20 years mapped the Japanese terms independently and selected one after discussion. Thus, for the first group, every MeSH UID was manually assigned to a Japanese term. The number of terms in the first group for which two or more different Japanese terms were assigned, the number of terms for which one term was assigned and the number of terms for which no term was assigned were 829, 150, and 60, respectively. We termed the dictionary including MeSH UIDs in the first group and Japanese terms assigned manually “curated.”

For the MeSH UIDs in the second group, if two or more Japanese terms are assigned, one Japanese term was automatically assigned according to the priority level of the six datasets. The list of the datasets in order of high to low priority was: KEGG Disease, KEGG Drug, JST thesaurus headwords, BLAH6 dictionary, HPO, and Wikidata. The highest priority was given to datasets manually curated by life-sciences experts, as described in the Discussion section. MeSH UIDs were then assigned to Japanese terms according to the priority of the datasets for MeSH UIDs such that at least one term was assigned using the six datasets. By combining the dictionary of the second group with the “curated” dictionary, we constructed a larger dictionary, which we termed “all.”

## Results

**Fig. 1.**
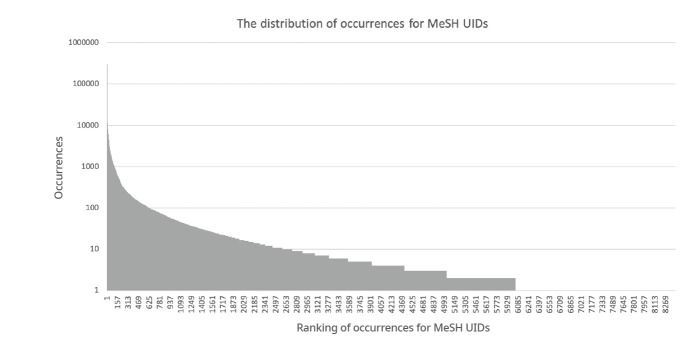
The distribution of occurrences of MeSH UIDs.

From LitCovid, which is annotated by Pubtator, 8419 MeSH UIDs were obtained. The total number of occurrences of the MeSH UIDs in LitCovid was 989,994. Fig. 1 shows the distribution of the number of occurrences of the MeSH UIDs. The x-axis shows the MeSH UIDs sorted by their occurrences, and the y-axis shows the number of occurrences on a logarithmic scale. As shown in Fig. 1, only a few words appeared very frequently and many words appeared only a few times.

Table 1 shows the number of terms assigned MeSH UIDs, the number of terms for the 8419 MeSH UIDs and the total occurrences of terms for the 989,994 occurrences in LitCovid in the six datasets.

There were no entries in KEGG Drug with MeSH UIDs; therefore, we assigned the MeSH UIDs to the Japanese terms that shared KEGG IDs with the English terms that exactly matched the MeSH entry terms, as explained in the Materials and Methods section.

As described in the Materials and Methods section, by using the Japanese terms obtained from datasets, we constructed “curated” and “all” dictionaries. The numbers of MeSH UIDs in the “curated” and “all” dictionaries were 1039 and 5805, respectively. Therefore, 12.34% and 68.95% of 8419 MeSH UIDs appearing in LitCovid were covered by the “curated” and “all” dictionaries. These numbers may seem quite small, especially for the “curated” dictionary. However, because the distribution of occurrences has a long tail, as shown in Fig. 1, and all MeSH UIDs with at least 50 occurrences were included in the “curated” dictionary, the “curated” and “all” dictionaries covered 94.63% and 98.99% of occurrences, respectively.

The “curated” dictionary is expected to be useful for text annotation because all Japanese terms in the dictionary were curated manually. Therefore, we uploaded it to the PubDictionaries system (https://pubdictionaries.org/) for wider use. The “all” dictionary may be useful in cases where more Japanese terms for MeSH UIDs are preferable. Both the “curated” and “all” dictionaries are available at https://github.com/acopom/blah7-dic under Creative Commons Attribution-ShareAlike 4.0 International license.

To check the quality of the dictionaries, we used some abstracts in LitCovid that also had Japanese translations by the authors. For example, the following sentence: “Cerebrovascular disease and vasculitis-related diseases have been reported as systemic complications of coronavirus disease 2019 (COVID-19),” which occurred in the abstract of PubMed ID 33051389, was annotated by PubTator as follows:

D002561: Cerebrovascular disease

D014657: vasculitis

C000657245: coronavirus disease 2019

C000657245: COVID-19

By using the “All” dictionary, we could translate these annotations as follows.

D002561: 脳血管障害

D014657: 脈管炎|血管炎

C000657245: 新型コロナウイルス感染症

The Japanese translation by the author for the sentence was “新型コロナウイルス感染症（COVID-19）蔓延に伴い，脳血管障害や血管炎関連疾患の合併が報告されるようになった。”, where the words in bold correspond to the translations of annotated words. Our dictionary assigned the same translations as the terms used by the author. In another example from PubMed ID 33051386, the sentence “Neuromuscular complications such as cerebrovascular disease, encephalopathy, meningoencephalitis, peripheral neuropathy, and myositis/myopathy have been reported to date.” was annotated by PubTator as follows.

D002561: cerebrovascular disease

D001927: encephalopathy

D008590: meningoencephalitis

D010523: peripheral neuropathy

D009220: myositis

D009135: myopathy

By using the “all” dictionary, we could translate these annotations as follows.

D002561: 脳血管障害

D001927: 脳疾患

D008590: 髄膜脳炎

D010523: 末梢神経系疾患

D009220: 筋炎

D009135: 筋疾患

The Japanese translation by the author for the sentence was “神経筋合併症としては，脳血管障害，脳症，髄膜脳炎，末梢神経障害，筋障害などが報告されている。”. In this case, although some terms in the sentence seem different from those of the dictionary, “脳症”/“脳疾患” and “末梢神経障害”/“末梢神経系障害” are synonyms. In addition, “筋障害” in the translation by the author is a broader concept than “筋炎” and “筋疾患” assigned by the dictionary. Therefore, we could confirm the quality of the dictionaries to some extent.

## Discussion

The six dictionaries we used have advantages and disadvantages. KEGG and JST contain curated data by experts and are reliable, but expert curation also involves the disadvantage of slow updates. In particular, JST is a dataset from 2015 that does not include new terms related to COVID-19. The same can be said for the BLAH6 dataset, which was based on dictionaries for machine translation in biology and medicine made by domain experts. Another feature of the BLAH6 dataset is that it contains duplicate spellings of the same word (in kanji and hiragana, for example). This is an advantage for use in applications such as assigning MeSH UIDs to Japanese texts, but for English-to-Japanese translations, another step is necessary to choose the standard spelling. In contrast, HPO and Wikidata are more up-to-date, but not as reliable as other dictionaries. The Japanese terms in HPO are machine translations of the English terms. Wikidata is written by human volunteers who are not guaranteed to be biomedical domain experts.

The MeSH UID for COVID-19 is C000657245 in MeSH 2020, which is also the case in the PubTator data we used for the experiments. However, in MeSH 2021, its status was updated from a Supplementary Concept to a Descriptor, and the UID is now D000086382. As shown by this example, NLM is quick to update MeSH, but it is difficult for official translations to keep up with this pace of MeSH updates. Thus, the methods for the quick and easy translation of terms will be necessary in events such as the current COVID-19 pandemic.

## Conclusion

We constructed two Japanese MeSH term dictionaries called “curated” and “all.” The numbers of MeSH UIDs in the “curated” and “all” dictionaries are 1039 and 5805, respectively. Although the numbers of MeSH UIDs may seem small, the “curated” and “all” dictionaries covered 94.63% and 98.99% of occurrences in LitCovid, respectively.

As discussed above, methods for quickly constructing a reliable dictionary of Japanese MeSH terms should be considered. To achieve this goal, methods of measuring and guaranteeing the reliability of translations of terms should be presented.

## Figures and Tables

**Table 1.** Japanese terms for MeSH UIDs obtained from each dataset

Dataset	No. of MeSH terms	Terms in LitCovid	Occurrences in LitCovid
Wikidata	15229	3565	887742
JST thesaurus headwords	15425	2026	353482
HPO	2176	1132	236892
KEGG Disease	2302	961	98616
KEGG Drug	-	1558	88118
BLAH6 dictionary	12771	2811	495494

MeSH, medical subject heading; UID, unique identifier; JST, Japan Science and Technology Agency; HPO, Human Phenotype Ontology; KEGG, Kyoto Encyclopedia of Genes and Genomes; BLAH6, 6th Biomedical Linked Annotation Hackathon.
